# Is Natural Language a Perigraphic Process? The Theorem about Facts and Words Revisited

**DOI:** 10.3390/e20020085

**Published:** 2018-01-26

**Authors:** Łukasz Dębowski

**Affiliations:** Institute of Computer Science, Polish Academy of Sciences, ul. Jana Kazimierza 5, 01-248 Warszawa, Poland; ldebowsk@ipipan.waw.pl; Tel.: +48-22-3800-553

**Keywords:** stationary processes, PPM code, mutual information, power laws, algorithmic information theory, natural language, 94A17, 60G10, 94A29, 68Q30, 68T50

## Abstract

As we discuss, a stationary stochastic process is nonergodic when a random persistent topic can be detected in the infinite random text sampled from the process, whereas we call the process strongly nonergodic when an infinite sequence of independent random bits, called probabilistic facts, is needed to describe this topic completely. Replacing probabilistic facts with an algorithmically random sequence of bits, called algorithmic facts, we adapt this property back to ergodic processes. Subsequently, we call a process perigraphic if the number of algorithmic facts which can be inferred from a finite text sampled from the process grows like a power of the text length. We present a simple example of such a process. Moreover, we demonstrate an assertion which we call the theorem about facts and words. This proposition states that the number of probabilistic or algorithmic facts which can be inferred from a text drawn from a process must be roughly smaller than the number of distinct word-like strings detected in this text by means of the Prediction by Partial Matching (PPM) compression algorithm. We also observe that the number of the word-like strings for a sample of plays by Shakespeare follows an empirical stepwise power law, in a stark contrast to Markov processes. Hence, we suppose that natural language considered as a process is not only non-Markov but also perigraphic.

## 1. Introduction

One of the motivating assumptions of information theory [1,2,3] is that communication in natural language can be reasonably modeled as a discrete stationary stochastic process, namely, an infinite sequence of discrete random variables with a well defined time-invariant probability distribution. The same assumption is made in several practical applications of computational linguistics, such as speech recognition [4] or part-of-speech tagging [5]. Whereas state-of-the-art stochastic models of natural language are far from being satisfactory, we may ask a more theoretically oriented question, namely:

What can be some general mathematical properties of natural language treated as a stochastic process, in view of empirical data?

In this paper, we will investigate a question of whether it is reasonable to assume that natural language communication is a *perigraphic* process.

To recall, a stationary process is called ergodic if the relative frequencies of all finite substrings in the infinite text generated by the process converge in the long run with probability one to some constants—the probabilities of the respective strings. Now, some basic linguistic intuition suggests that natural language does not satisfy this property, cf. ([3], Section 6.4). Namely, we can probably agree that there is a variation of topics of texts in natural language, and these topics can be empirically distinguished by counting relative frequencies of certain substrings called keywords. Hence, we expect that the relative frequencies of keywords in a randomly selected text in natural language are random variables depending on the random text topic. In the limit, for an infinitely long text, we may further suppose that the limits of relative frequencies of keywords persist to be random, and if this is true then natural language is not ergodic, i.e., it is nonergodic.

In this paper, we will entertain first a stronger hypothesis, namely, that natural language communication is strongly nonergodic. Informally speaking, a stationary process will be called strongly nonergodic if its random persistent topic has to be described using an infinite sequence of probabilistically independent binary random variables, called probabilistic facts. Like nonergodicity, strong nonergodicity is not empirically verifiable if we only have a single infinite sequence of data. However, replacing probabilistic facts with an algorithmically random sequence of bits, called algorithmic facts, we can adapt the property of strong nonergodicity back to ergodic processes. Subsequently, we will call a process *perigraphic* if the number of algorithmic facts which can be inferred from a finite text sampled from the process grows like a power of the text length. It is a general observation that perigraphic processes have uncomputable distributions.

It is interesting to note that *perigraphic* processes can be singled out by some statistical properties of the texts they generate. We will exhibit a proposition, which we call the theorem about facts and words. Suppose that we have a finite text drawn from a stationary process. The theorem about facts and words says that the number of independent probabilistic or algorithmic facts that can be reasonably inferred from the text must be roughly smaller than the number of distinct word-like strings detected in the text by some standard data compression algorithm called the Prediction by Partial Matching (PPM) code [6,7]. It is important to stress that in this theorem we do not relate the numbers all facts and all word-like strings, which would sound trivial, but we compare only the numbers of independent facts and distinct word-like strings.

Having the theorem about facts and words, we can also discuss some empirical data. Since the number of distinct word-like strings for texts in natural language follows an empirical stepwise power law, in a stark contrast to Markov processes, consequently, we suppose that the number of inferrable random facts for natural language also follows a power law. That is, we suppose that natural language is not only non-Markov but also *perigraphic*.

Whereas in this paper we fill several important missing gaps and provide an overarching narration, the basic ideas presented in this paper are not so new. The starting point was a corollary of Zipf’s law and a hypothesis by Hilberg. Zipf’s law is an empirical observation that in texts in natural language, the frequencies of words obey a power law decay when we sort the words according to their decreasing frequencies [8,9]. A corollary of this law, called Heaps’ law [10,11,12,13], states that the number of distinct words in a text in natural language grows like a power of the text length. In contrast to these simple empirical observations, Hilberg’s hypothesis is a less known conjecture about natural language that the entropy of a text chunk of an increasing length [14] or the mutual information between two adjacent text chunks [15,16,17,18] obey also a power law growth. In Ref. [19], it was heuristically shown that, if Hilberg’s hypothesis for mutual information is satisfied for an arbitrary stationary stochastic process, then texts drawn from this process satisfy also a kind of Heaps’ law if we detect the words using the grammar-based codes [20,21,22,23]. This result is a historical antecedent of the theorem about facts and words.

Another important step was a discovery of some simple strongly nonergodic processes, satisfying the power law growth of mutual information, called Santa Fe processes, discovered by Dębowski in August 2002, but first reported only in [24]. Subsequently, in Ref. [25], a completely formal proof of the theorem about facts and words for strictly minimal grammar-based codes [23,26] was provided. The respective related theory of natural language was later reviewed in [27,28] and supplemented by a discussion of Santa Fe processes in [29]. A drawback of this theory at that time was that strictly minimal grammar-based codes used in the statement of the theorem about facts and words are not computable in a polynomial time [26]. This precluded an empirical verification of the theory.

To state the relative novelty, in this paper, we are glad to announce a new stronger version of the theorem about facts and words for a somewhat more elegant definition of inferrable facts and the PPM code, which is computable almost in a linear time. For the first time, we also present two cases of the theorem: one for strongly nonergodic processes, applying Shannon information theory, and one for general stationary processes, applying algorithmic information theory. Having these results, we can supplement them finally with a rudimentary discussion of some empirical data.

The organization of this paper is as follows. In Section 2, we discuss some properties of ergodic and nonergodic processes. In Section 3, we define strongly nonergodic processes and we present some examples of them. Analogically, in Section 4, we discuss perigraphic processes. In Section 5, we discuss two versions of the theorem about facts and words. In Section 6, we discuss some empirical data and we suppose that natural language may be a perigraphic process. In Section 7, we offer concluding remarks. Moreover, three appendices follow the body of the paper. In Appendix A, we prove the first part of the theorem about facts and words. In Appendix B, we prove the second part of this theorem. In Appendix C, we show that that the number of inferrable facts for the Santa Fe processes follows a power law.

## 2. Ergodic and Nonergodic Processes

We assume that the reader is familiar with some probability measure theory [30]. For a real-valued random variable *Y* on a probability space (Ω,J,P), we denote its expectation

(1) $$\begin{array}{c} E\;Y:=\int YdP. \end{array}$$

Consider now a discrete stochastic process ${\left(X_{i}\right)}_{i=1}^{\infty }=\left(X_{1},\;X_{2},\;\ldots \right)$, where random variables $X_{i}$ take values from a set X of countably many distinct symbols, such as letters with which we write down texts in natural language. We denote blocks of consecutive random variables $X_{j}^{k}:=\left(X_{j},\ldots ,X_{k}\right)$ and symbols $x_{j}^{k}:=\left(x_{j},\ldots ,x_{k}\right)$. Let us define a binary random variable telling whether some string $x_{1}^{n}$ has occurred in sequence ${\left(X_{i}\right)}_{i=1}^{\infty }$ on positions from *i* to i+n−1,

(2) $$\begin{array}{c} \Phi_{i}\left(x_{1}^{n}\right):=1\left\{X_{i}^{i+n-1}=x_{1}^{n}\right\}, \end{array}$$

where

(3) $$\begin{array}{c} 1\left\{\phi \right\}=\left\{\begin{array}{cc} 1, & \mathrm{if}\;\phi \;\mathrm{is}\;\mathrm{true},\\ 0, & \mathrm{if}\;\phi \;\mathrm{is}\;\mathrm{false}. \end{array}\right. \end{array}$$

The expectation of this random variable,

(4) $$\begin{array}{c} E\;\Phi_{i}\left(x_{1}^{n}\right)=P\left(X_{i}^{i+n-1}=x_{1}^{n}\right), \end{array}$$

is the probability of the chosen string according to the considered probability measure *P*, whereas the arithmetic average of consecutive random variables $\frac{1}{m}\sum_{i=1}^{m}\Phi_{i}\left(x_{1}^{n}\right)$ is the relative frequency of the same string in a finite sequence of random symbols $X_{1}^{m+n-1}$.

Process ${\left(X_{i}\right)}_{i=1}^{\infty }$ is called *stationary* (with respect to a probability measure *P*) if expectations $E\;\Phi_{i}\left(x_{1}^{n}\right)$ do not depend on position *i* for any string $x_{1}^{n}$. In this case, we have the following well known theorem, which establishes that the limiting relative frequencies of strings $x_{1}^{n}$ in infinite sequence ${\left(X_{i}\right)}_{i=1}^{\infty }$ exist almost surely, i.e., with probability 1:

**Theorem** **1** (ergodic theorem, cf. e.g., [31])**.** *For any discrete stationary process ${\left(X_{i}\right)}_{i=1}^{\infty }$, there exist limits*

(5) $$\begin{array}{c} \Phi \left(x_{1}^{n}\right):=\lim_{m\to \infty }\frac{1}{m}\sum_{i=1}^{m}\Phi_{i}\left(x_{1}^{n}\right)\;\mathrm{almost}\;\mathrm{surely}, \end{array}$$

*with expectations $E\;\Phi \left(x_{1}^{n}\right)=E\;\Phi_{i}\left(x_{1}^{n}\right)$.*

In general, limits $\Phi (x_{1}^{n})$ are random variables depending on a particular value of infinite sequence ${\left(X_{i}\right)}_{i=1}^{\infty }$. It is quite natural, however, to require that the relative frequencies of strings $\Phi (x_{1}^{n})$ are almost surely constants, equal to the expectations $E\;\Phi_{i}\left(x_{1}^{n}\right)$. Subsequently, process ${\left(X_{i}\right)}_{i=1}^{\infty }$ will be called *ergodic* (with respect to a probability measure *P*) if limits $\Phi (x_{1}^{n})$ are almost surely constant for any string $x_{1}^{n}$. The standard definition of an ergodic process is more abstract but is equivalent to this statement ([31], Lemma 7.15).

The following examples of ergodic processes are well known:

1. Process ${\left(X_{i}\right)}_{i=1}^{\infty }$ is called *IID* (independent identically distributed) if
  (6) $$\begin{array}{c} P\left(X_{1}^{n}=x_{1}^{n}\right)=\pi \left(x_{1}\right)\ldots \pi \left(x_{n}\right). \end{array}$$
  All IID processes are ergodic.
2. Process ${\left(X_{i}\right)}_{i=1}^{\infty }$ is called *Markov* (of order 1) if
  (7) $$\begin{array}{c} P\left(X_{1}^{n}=x_{1}^{n}\right)=\pi \left(x_{1}\right)p\left(x_{2}|x_{1}\right)\ldots p\left(x_{n}|x_{n-1}\right). \end{array}$$
  A Markov process is ergodic in particular if
  (8) $$\begin{array}{c} p(x_{i}|x_{i-1})>c>0. \end{array}$$
  For a sufficient and necessary condition, see ([32], Theorem 7.16).
3. Process ${\left(X_{i}\right)}_{i=1}^{\infty }$ is called *hidden Markov* if $X_{i}=g\left(S_{i}\right)$ for a certain Markov process ${\left(S_{i}\right)}_{i=1}^{\infty }$ and a function *g*. A hidden Markov process is ergodic in particular if the underlying Markov process is ergodic.

Whereas IID and Markov processes are some basic models in probability theory, hidden Markov processes are of practical importance in computational linguistics [4,5]. Hidden Markov processes as considered there usually satisfy condition (8) and therefore they are ergodic.

Let us call a probability measure *P* stationary or ergodic, respectively, if the process ${\left(X_{i}\right)}_{i=1}^{\infty }$ is stationary or ergodic with respect to the measure *P*. Suppose that we have a stationary measure *P* that generates some data ${\left(X_{i}\right)}_{i=1}^{\infty }$. We can define a new random measure *F* equal to the relative frequencies of blocks in the data ${\left(X_{i}\right)}_{i=1}^{\infty }$. It turns out that the measure *F* is almost surely ergodic. Formally, we have this proposition.

**Theorem** **2** (cf. ([33], Theorem 9.10))**.** *Any process ${\left(X_{i}\right)}_{i=1}^{\infty }$ with a stationary measure P is almost surely ergodic with respect to the random measure F given by*

(9) $$\begin{array}{c} F\left(X_{1}^{n}=x_{1}^{n}\right):=\Phi \left(x_{1}^{n}\right). \end{array}$$

Moreover, from the random measure *F*, we can obtain the stationary measure *P* by integration, $P\left(X_{1}^{n}=x_{1}^{n}\right)=E\;F\left(X_{1}^{n}=x_{1}^{n}\right)$. The following result asserts that this integral representation of measure *P* is unique.

**Theorem** **3** (ergodic decomposition, cf. ([33], Theorem 9.12))**.** *Any stationary probability measure P can be represented as*

(10) $$\begin{array}{c} P\left(X_{1}^{n}=x_{1}^{n}\right)=\int F\left(X_{1}^{n}=x_{1}^{n}\right)d\nu \left(F\right), \end{array}$$

*where ν is a unique measure on stationary ergodic measures.*

In other words, stationary ergodic measures are some building blocks from which we can construct any stationary measure. For a stationary probability measure *P*, the particular values of the random ergodic measure *F* are called the ergodic components of measure *P*.

Consider for instance, a Bernoulli(θ) process with measure

(11) $$\begin{array}{c} F_{\theta }\left(X_{1}^{n}=x_{1}^{n}\right)=\theta^{\sum_{i=1}^{n}x_{i}}{\left(1-\theta \right)}^{n-\sum_{i=1}^{n}x_{i}}, \end{array}$$

where $x_{i}\in \left\{0,1\right\}$ and θ∈[0,1]. This measure will be contrasted with the measure of a mixture Bernoulli process with parameter θ uniformly distributed on interval [0,1],

(12) P(X1n=x1n)=∫01Fθ(X1n=x1n)dθ=1n+1n∑i=1nxi−1.

Measure (11) is a measure of an IID process and is therefore ergodic, whereas measure (12) is a mixture of ergodic measures and hence it is nonergodic.

## 3. Strongly Nonergodic Processes

According to our definition, a process is ergodic when the relative frequencies of any strings in a random sample in the long run converge to some constants. Consider now the following thought experiment. Suppose that we select a random book from a library. In [34], it was observed that there is hardly any book that contains both the word *lemma* and the word *love*, namely, there are some keywords that are specific to particular topics of texts. We can pursue this idea one little step farther. Counting the relative frequencies of keywords, such as *lemma* for a text on mathematics and *love* for a romance, we can effectively recognize the topic of the book. Simply put, the relative frequencies of some keywords will be higher for books concerning some topics, whereas they will be lower for books concerning other topics. Hence, in our thought experiment, we expect that the relative frequencies of keywords are some random variables with values depending on the particular topic of the randomly selected book. Since keywords are just some particular strings, we may conclude that the stochastic process that models natural language should be nonergodic.

The above thought experiment provides another perspective onto nonergodic processes. According to the following theorem, a process is nonergodic when we can effectively distinguish in the limit at least two random topics in it. In the statement, function $f:X^{*}\to \left\{0,1,2\right\}$ assumes values 0 or 1 when we can identify the topic, whereas it takes value 2 when we are not certain which topic a given text is about.

**Theorem** **4** (cf. [24])**.** A stationary discrete process ${\left(X_{i}\right)}_{i=1}^{\infty }$ is nonergodic if and only if there exists a function $f:X^{*}\to \left\{0,1,2\right\}$ and a binary random variable Z such that 0<P(Z=0)<1 and

(13) $$\begin{array}{c} \lim_{n\to \infty }P\left(f\left(X_{i}^{i+n-1}\right)=Z\right)=1 \end{array}$$

*for any position i∈N.*

A binary variable *Z* satisfying condition (13) will be called a *probabilistic fact*. A probabilistic fact tells which of two topics the infinite text generated by the stationary process is about. It is a kind of a random switch which is preset before we start scanning the infinite text; compare a similar wording in [35]. To keep the proofs simple, here we only give a new elementary proof of the “⇒” statement of Theorem 4. The proof of the “⇐” part applies some measure theory and follows the idea of Theorem 9 from [24] for strongly nonergodic processes, which we will discuss in the next paragraph.

**Proof.**  (only ⇒) Suppose that process ${\left(X_{i}\right)}_{i=1}^{\infty }$ is nonergodic. Then, there exists a string $x_{1}^{k}$ such that Φ≠EΦ for $\Phi :=\Phi (x_{1}^{k})$ with some positive probability. Hence, there exists a real number *y* such that P(Φ=y)=0 and

(14) $$\begin{array}{c} P(\Phi >y)=1-P(\Phi <y)\in (0,1). \end{array}$$

Define $Z:=1\left\{\Phi >y\right\}$ and $f\left(X_{i}^{i+n-1}\right):=Z_{in}:=1\left\{\Phi_{in}>y\right\}$, where

(15) $$\begin{array}{c} \Phi_{in}:=\frac{1}{n-k+1}\sum_{j=i}^{i+n-k}\Phi_{j}\left(x_{1}^{k}\right). \end{array}$$

Since $\lim_{n\to \infty }\Phi_{in}=\Phi$ almost surely and Φ satisfies (14), convergence $\lim_{n\to \infty }Z_{in}=Z$ also holds almost surely. Applying the Lebesgue dominated convergence theorem, we obtain

(16) $$\begin{array}{cc} \lim_{n\to \infty }P\left(f\left(X_{i}^{i+n-1}\right)=Z\right) & =\lim_{n\to \infty }E\;\left[Z_{in}Z+\left(1-Z_{in}\right)\left(1-Z\right)\right]\\ & =E\;\left[Z^{2}+{\left(1-Z\right)}^{2}\right]=1. \end{array}$$

 ☐

As for books in the natural language, we may have an intuition that the pool of available book topics is extremely large and contains many more topics than just two. For this reason, we may need not a single probabilistic fact *Z* but rather a sequence of probabilistic facts $Z_{1},\;Z_{2},\;\ldots$ to specify the topic of a random book completely. Formally, stationary processes requiring an infinite sequence of independent uniformly distributed probabilistic facts to describe the topic of an infinitely long text will be called strongly nonergodic.

**Definition** **1** (cf. [24,25])**.** *A stationary discrete process ${\left(X_{i}\right)}_{i=1}^{\infty }$ is called* strongly nonergodic if there exist a function $g:N\times X^{*}\to \left\{0,1,2\right\}$ and a binary IID process ${\left(Z_{k}\right)}_{k=1}^{\infty }$ such that $P\left(Z_{k}=0\right)=P\left(Z_{k}=1\right)=1/2$ and

(17) $$\begin{array}{c} \lim_{n\to \infty }P\left(g\left(k;X_{i}^{i+n-1}\right)=Z_{k}\right)=1 \end{array}$$

*for any position i∈N and any index k∈N.*

As we have stated above, for a strongly nonergodic process, there is an infinite number of independent probabilistic facts ${\left(Z_{k}\right)}_{k=1}^{\infty }$ with a uniform distribution on the set $\left\{0,1\right\}$. Formally, these probabilistic facts can be assembled into a single real random variable $T=\sum_{k=1}^{\infty }2^{-k}Z_{k}$, which is uniformly distributed on the unit interval [0,1]. The value of variable *T* identifies the topic of a random infinite text generated by the stationary process. Thus, for a strongly nonergodic process, we have a continuum of available topics which can be incrementally identified from any sufficiently long text. Put formally, according to Theorem 9 from [24], a stationary process is strongly nonergodic if and only if its shift-invariant σ-field contains a nonatomic sub-σ-field. We note in passing that in [24] strongly nonergodic processes were called *uncountable description processes*.

In view of Theorem 9 from [24], the mixture Bernoulli process (12) is some example of a strongly nonergodic process. In this case, the parameter θ plays the role of the random variable $T=\sum_{k=1}^{\infty }2^{-k}Z_{k}$. Showing that condition (17) is satisfied for this process in an elementary fashion is a tedious exercise. Hence, let us present now a simpler guiding example of a strongly nonergodic process, which we introduced in [24,25] and called the Santa Fe process. Let ${\left(Z_{k}\right)}_{k=1}^{\infty }$ be a binary IID process with $P\left(Z_{k}=0\right)=P\left(Z_{k}=1\right)=1/2$. Let ${\left(K_{i}\right)}_{i=1}^{\infty }$ be an IID process with $K_{i}$ assuming values in natural numbers with a power-law distribution

(18) $$\begin{array}{c} P\left(K_{i}=k\right)\propto \frac{1}{k^{\alpha }},\;\alpha >1. \end{array}$$

The *Santa Fe process* with exponent α is a sequence ${\left(X_{i}\right)}_{i=1}^{\infty }$, where

(19) $$\begin{array}{c} X_{i}=\left(K_{i},Z_{K_{i}}\right) \end{array}$$

are pairs of a random number $K_{i}$ and the corresponding probabilistic fact $Z_{K_{i}}$. The Santa Fe process is strongly nonergodic since condition (17) holds for example for

(20) $$\begin{array}{c} g\left(k;x_{1}^{n}\right)=\left\{\begin{array}{cc} 0, & \mathrm{if}\;\mathrm{for}\;\mathrm{all}\;1\leq i\leq n,\;x_{i}=\left(k,z\right)\Rightarrow x_{i}=\left(k,0\right),\\ 1, & \mathrm{if}\;\mathrm{for}\;\mathrm{all}\;1\leq i\leq n,\;x_{i}=\left(k,z\right)\Rightarrow x_{i}=\left(k,1\right),\\ 2, & \mathrm{else}. \end{array}\right. \end{array}$$

Simply speaking, function g(k;·) returns 0 or 1 when an unambiguous value of the second constituent can be read off from pairs $x_{i}=\left(k,\cdot \right)$ and returns 2 when there is some ambiguity. Condition (17) is satisfied since

(21) $$\begin{array}{cc} P(g\left(k;X_{i}^{i+n-1}\right)=Z_{k}) & =P(K_{i}=k\;\mathrm{for}\;\mathrm{some}\;1\leq i\leq n)\\ & =1-{\left(1-P\left(K_{i}=k\right)\right)}^{n}\underset{n\to \infty }{\to }1. \end{array}$$

Some salient property of the Santa Fe process is the power law growth of the expected number of probabilistic facts, which can be inferred from a finite text drawn from the process. Consider a strongly nonergodic process ${\left(X_{i}\right)}_{i=1}^{\infty }$. The set of initial independent probabilistic facts inferrable from a finite text $X_{1}^{n}$ will be defined as

(22) $$\begin{array}{c} U\left(X_{1}^{n}\right):=\left\{l\in N:g\left(k;X_{1}^{n}\right)=Z_{k}\;\mathrm{for}\;\mathrm{all}\;k\leq l\right\}. \end{array}$$

In other words, we have $U\left(X_{1}^{n}\right)=\left\{1,2,\ldots ,l\right\}$, where *l* is the largest number such that $g\left(k;X_{1}^{n}\right)=Z_{k}$ for all k≤l. To capture the power-law growth of an arbitrary function s:N→R, we will denote the Hilberg exponent defined

(23) $$\begin{array}{c} \underset{n\to \infty }{\mathrm{hilb}}s\left(n\right):=\underset{n\to \infty }{\lim\;\sup}\frac{\log^{+}s\left(2^{n}\right)}{\log2^{n}}, \end{array}$$

where $\log^{+}x:=\log\left(x+1\right)$ for x≥0 and $\log^{+}x:=0$ for x<0, cf. [36]. In contrast to Ref. [36], for technical reasons, we define the Hilberg exponent only for an exponentially sparse subsequence of terms $s(2^{n})$ rather than all terms s(n). Moreover, in [36], the Hilberg exponent was considered only for mutual information $s\left(n\right)=I\left(X_{1}^{n};X_{n+1}^{2n}\right)$, defined later in Equation (51). We observe that for the exact power law growth $s\left(n\right)=n^{\beta }$ with β≥0 we have ${\mathrm{hilb}}_{n\to \infty }s\left(n\right)=\beta$. More generally, the Hilberg exponent captures an asymptotic power-law growth of the sequence. As shown in Appendix C, for the Santa Fe process with exponent α, we have the asymptotic power-law growth

(24) $$\begin{array}{c} \underset{n\to \infty }{\mathrm{hilb}}E\;\mathrm{card}U\left(X_{1}^{n}\right)=1/\alpha \in \left(0,1\right). \end{array}$$

This property distinguishes the Santa Fe process from the mixture Bernoulli process (12), for which the respective Hilberg exponent is zero, as we discuss in Section 6.

## 4. Perigraphic Processes

Is it possible to demonstrate by a statistical investigation of texts that natural language is really strongly nonergodic and satisfies a condition similar to (24)? In the thought experiment described in the beginning of the previous section, we have ignored the issue of constructing an infinitely long text. In reality, every book with a well defined topic is finite. If we want to obtain an unbounded collection of texts, we need to assemble a corpus of different books and it depends on our assembling criteria whether the books in the corpus will concern some persistent random topic. Moreover, if we already have a *single* infinite sequence of books generated by some stationary source and we estimate probabilities as relative frequencies of blocks of symbols in this sequence, then, by Theorem 2, we will obtain an ergodic probability measure almost surely.

In this situation, we may ask whether the idea of the power-law growth of the number of inferrable probabilistic facts can be translated somehow to the case of ergodic measures. Some straightforward method to apply is to replace the sequence of independent uniformly distributed probabilistic facts ${\left(Z_{k}\right)}_{k=1}^{\infty }$, being random variables, with an algorithmically random sequence of particular binary digits ${\left(z_{k}\right)}_{k=1}^{\infty }$. Such digits $z_{k}$ will be called *algorithmic facts* in contrast to variables $Z_{k}$ being called *probabilistic facts*.

Let us recall some basic concepts. For a discrete random variable *X*, let P(X) denote the random variable that takes value P(X=x) when *X* takes value *x*. We will introduce the pointwise entropy

(25) $$\begin{array}{c} H(X):=-\log P(X), \end{array}$$

where log stands for the natural logarithm. The prefix-free Kolmogorov complexity K(u) of a string *u* is the length of the shortest self-delimiting program written in binary digits that prints out string *u* ([37], Chapter 3). K(u) is the founding concept of the algorithmic information theory and is an analogue of the pointwise entropy. To keep our notation analogical to (25), we will write the algorithmic entropy

(26) $$\begin{array}{c} H_{a}\left(u\right):=K\left(u\right)\log2. \end{array}$$

If the probability measure is computable, then the algorithmic entropy is close to the pointwise entropy. On the one hand, by the Shannon–Fano coding for a computable probability measure, the algorithmic entropy is less than the pointwise entropy plus a constant which depends on the probability measure and the dimensionality of the distribution ([37], Corollary 4.3.1). Formally,

(27) $$\begin{array}{c} H_{a}\left(X_{1}^{n}\right)\leq H\left(X_{1}^{n}\right)+2\log n+C_{P}, \end{array}$$

where $C_{P}\geq 0$ is a certain constant depending on the probability measure *P*. On the other hand, since the prefix-free Kolmogorov complexity is also the length of a prefix-free code, we have

(28) $$\begin{array}{c} E\;H_{a}\left(X_{1}^{n}\right)\geq E\;H\left(X_{1}^{n}\right). \end{array}$$

It is also true that $H_{a}\left(X_{1}^{n}\right)\geq H\left(X_{1}^{n}\right)$ for sufficiently large *n* almost surely ([38], Theorem 3.1). Thus, we have shown that the algorithmic entropy is in some sense close to the pointwise entropy, for a computable probability measure.

Next, we will discuss the difference between probabilistic and algorithmic randomness. Whereas for an IID sequence of random variables ${\left(Z_{k}\right)}_{k=1}^{\infty }$ with $P\left(Z_{k}=0\right)=P\left(Z_{k}=1\right)=1/2$ we have

(29) $$\begin{array}{c} H(Z_{1}^{k})=k\log2, \end{array}$$

similarly an infinite sequence of binary digits ${\left(z_{k}\right)}_{k=1}^{\infty }$ is called algorithmically random (in the Martin-Löf sense) when there exists a constant C≥0 such that

(30) $$\begin{array}{c} H_{a}\left(z_{1}^{k}\right)\geq k\log2-C \end{array}$$

for all k∈N ([37], Theorem 3.6.1). The probability that the aforementioned sequence of random variables ${\left(Z_{k}\right)}_{k=1}^{\infty }$ is algorithmically random equals 1—for example by ([38], Theorem 3.1), so algorithmically random sequences are typical realizations of sequence ${\left(Z_{k}\right)}_{k=1}^{\infty }$.

Let ${\left(X_{i}\right)}_{i=1}^{\infty }$ be a stationary process. We observe that generalizing condition (17) in an algorithmic fashion does not make much sense. Namely, condition

(31) $$\begin{array}{c} \lim_{n\to \infty }P\left(g\left(k;X_{i}^{i+n-1}\right)=z_{k}\right)=1 \end{array}$$

is trivially satisfied for any stationary process for a certain computable function $g:N\times X^{*}\to \left\{0,1,2\right\}$ and an algorithmically random sequence ${\left(z_{k}\right)}_{k=1}^{\infty }$. It turns out so since there exists a computable function $\omega :N\times N\to \left\{0,1\right\}$ such that $\lim_{n\to \infty }\omega \left(k;n\right)=\Omega_{k}$, where ${\left(\Omega_{k}\right)}_{k=1}^{\infty }$ is the binary expansion of the halting probability $\Omega =\sum_{k=1}^{\infty }2^{-k}\Omega_{k}$, which is a lower semi-computable algorithmically random sequence ([37], Section 3.6.2).

In spite of this negative result, the power-law growth of the number of inferrable algorithmic facts corresponds to some nontrivial property. For a computable function $g:N\times X^{*}\to \left\{0,1,2\right\}$ and an algorithmically random sequence of binary digits ${\left(z_{k}\right)}_{k=1}^{\infty }$, which we will call *algorithmic facts*, the set of initial algorithmic facts inferrable from a finite text $X_{1}^{n}$ will be defined as

(32) $$\begin{array}{c} U_{a}\left(X_{1}^{n}\right):=\left\{l\in N:g\left(k;X_{1}^{n}\right)=z_{k}\;\mathrm{for}\;\mathrm{all}\;k\leq l\right\}. \end{array}$$

Subsequently, we will call a process perigraphic if the expected number of algorithmic facts which can be inferred from a finite text sampled from the process grows asymptotically like a power of the text length.

**Definition** **2.** *A stationary discrete process ${\left(X_{i}\right)}_{i=1}^{\infty }$ is called* perigraphic *if*

(33) $$\begin{array}{c} \underset{n\to \infty }{\mathrm{hilb}}E\;\mathrm{card}\;U_{a}\left(X_{1}^{n}\right)>0 \end{array}$$

for some computable function $g:N\times X^{*}\to \left\{0,1,2\right\}$ and an algorithmically random sequence of binary digits ${\left(z_{k}\right)}_{k=1}^{\infty }$.

Perigraphic processes can be ergodic. The proof of Theorem A10 from Appendix C can be easily adapted to show that some example of a perigraphic process is the Santa Fe process with sequence ${\left(Z_{k}\right)}_{k=1}^{\infty }$ replaced by an algorithmically random sequence of binary digits ${\left(z_{k}\right)}_{k=1}^{\infty }$. To be very concrete, the example of a perigraphic process can be process ${\left(X_{i}\right)}_{i=1}^{\infty }$ with

(34) $$\begin{array}{c} X_{i}=\left(K_{i},\Omega_{K_{i}}\right) \end{array}$$

where ${\left(\Omega_{k}\right)}_{k=1}^{\infty }$ is the binary expansion of the halting probability and ${\left(K_{i}\right)}_{i=1}^{\infty }$ is an IID process with $K_{i}$ assuming values in natural numbers with the power-law distribution (18). This process is not only perigraphic but also IID and hence ergodic.

We can also easily show the following proposition.

**Theorem** **5.** Any perigraphic process ${\left(X_{i}\right)}_{i=1}^{\infty }$ has an uncomputable measure P.

**Proof.**  Assume that a perigraphic process ${\left(X_{i}\right)}_{i=1}^{\infty }$ has a computable measure *P*. By inequalities (A25) and (A26) from Appendix A, we have

(35) $$\begin{array}{cc} \underset{n\to \infty }{\mathrm{hilb}}E\;\mathrm{card}U_{a}\left(X_{1}^{n}\right) & \leq \underset{n\to \infty }{\mathrm{hilb}}E\;\left[H_{a}\left(X_{1}^{n}\right)-H\left(X_{1}^{n}\right)\right]. \end{array}$$

Since, for a computable measure *P*, we also have inequality (27), then

(36) $$\begin{array}{c} \underset{n\to \infty }{\mathrm{hilb}}E\;\mathrm{card}\;U_{a}\left(X_{1}^{n}\right)=0. \end{array}$$

Since we have obtained a contradiction with the assumption that the process is perigraphic, measure *P* cannot be computable. ☐

## 5. Theorem about Facts and Words

In this section, we will present a result about stationary processes, which we call the theorem about facts and words. This proposition states that the expected number of independent probabilistic or algorithmic facts inferrable from the text drawn from a stationary process must be roughly less than the expected number of distinct word-like strings detectable in the text by a simple procedure involving the PPM compression algorithm. This result states, in particular, that an asymptotic power law growth of the number of inferrable probabilistic or algorithmic facts as a function of the text length produces a statistically measurable effect, namely, an asymptotic power law growth of the number of word-like strings.

To state the theorem about facts and words formally, we need first to discuss the PPM code. The general idea of the PPM code comes from Refs. [6,7], developed independently. This compression scheme was called the PPM code in [7], which stands for “Prediction by Partial Matching” and prevails in the literature, whereas it was called measure R in [6,39]. Whereas Ref. [7] focused on practical applications to data compression and earned most of the fame, in Refs. [6,39], one can find a few results that matter for theoretical considerations. Let us denote strings of symbols $x_{j}^{k}:=\left(x_{j},\ldots ,x_{k}\right)$, adopting an important convention that $x_{j}^{k}$ is the empty string for k<j. In the following, we consider strings over a finite alphabet, say, $x_{i}\in X=\left\{1,\ldots ,D\right\}$. We define the frequency of a substring $w_{1}^{k}$ in a string $x_{1}^{n}$ as

(37) $$\begin{array}{c} N\left(w_{1}^{k}|x_{1}^{n}\right):=\sum_{i=1}^{n-k+1}1\left\{x_{i}^{i+k-1}=w_{1}^{k}\right\}. \end{array}$$

Now, we will define the PPM probabilities in a way that is closer to the conventions of paper [6,39] than to the conventions of Ref. [7]. In particular, in Equation (38), we consider frequencies of strings $x_{i-k}^{i}$ and $x_{i-k}^{i-1}$ in different strings, $x_{1}^{i-1}$ and $x_{1}^{i-2}$, respectively, in the numerator and in the denominator to guarantee the proper normalization according to our definition of $N(w_{1}^{k}|x_{1}^{n})$.

**Definition** **3** (cf. [6,7])**.** *For $x_{1}^{n}\in X^{n}$ and $k\in \left\{-1,0,1,\ldots \right\}$, we put*

(38) $$\begin{array}{cc} {\mathrm{PPM}}_{k}\left(x_{i}|x_{1}^{i-1}\right) & :=\left\{\begin{array}{cc} \frac{1}{D}, & i\leq k,\\ \frac{N(x_{i-k}^{i}|x_{1}^{i-1})+1}{N(x_{i-k}^{i-1}|x_{1}^{i-2})+D}, & i>k. \end{array}\right. \end{array}$$

*Quantity ${\mathrm{PPM}}_{k}\left(x_{i}|x_{1}^{i-1}\right)$ is called the* conditional PPM probability *of order k of symbol $x_{i}$ given string $x_{1}^{i-1}$. Next, we put*

(39) $$\begin{array}{cc} {\mathrm{PPM}}_{k}\left(x_{1}^{n}\right) & :=\prod_{i=1}^{n}{\mathrm{PPM}}_{k}\left(x_{i}|x_{1}^{i-1}\right). \end{array}$$

*Quantity ${\mathrm{PPM}}_{k}\left(x_{1}^{n}\right)$ is called the* PPM probability *of order k of string $x_{1}^{n}$. Finally, we put*

(40) $$\begin{array}{cc} \mathrm{PPM}(x_{1}^{n}) & :=\frac{6}{\pi^{2}}\sum_{k=-1}^{\infty }\frac{{\mathrm{PPM}}_{k}\left(x_{1}^{n}\right)}{{\left(k+2\right)}^{2}}. \end{array}$$

*Quantity $\mathrm{PPM}(x_{1}^{n})$ is called the (total)* PPM probability *of the string $x_{1}^{n}$.*

Quantity ${\mathrm{PPM}}_{k}\left(x_{1}^{n}\right)$ is an incremental approximation of the unknown true probability of the string $x_{1}^{n}$, assuming that the string has been generated by a Markov process of order *k*. In contrast, quantity $\mathrm{PPM}(x_{1}^{n})$ is a mixture of such Markov approximations for all finite orders. In general, the PPM probabilities are probability distributions over strings of a fixed length. That is:

- ${\mathrm{PPM}}_{k}\left(x_{i}|x_{1}^{i-1}\right)>0$ and $\sum_{x_{i}\in X}{\mathrm{PPM}}_{k}\left(x_{i}|x_{1}^{i-1}\right)=1$,
- ${\mathrm{PPM}}_{k}\left(x_{1}^{n}\right)>0$ and $\sum_{x_{1}^{n}\in X^{n}}{\mathrm{PPM}}_{k}\left(x_{1}^{n}\right)=1$,
- $\mathrm{PPM}(x_{1}^{n})>0$ and $\sum_{x_{1}^{n}\in X^{n}}\mathrm{PPM}\left(x_{1}^{n}\right)=1$.

In the following, we define an analogue of the pointwise entropy

(41) $$\begin{array}{cc} H_{\mathrm{PPM}}\left(x_{1}^{n}\right) & :=-\log\mathrm{PPM}(x_{1}^{n}). \end{array}$$

Quantity $H_{\mathrm{PPM}}\left(x_{1}^{n}\right)$ will be called the length of the PPM code for the string $x_{1}^{n}$. By nonnegativity of the Kullback–Leibler divergence, we have for any random block $X_{1}^{n}$ that

(42) $$\begin{array}{cc} E\;H_{\mathrm{PPM}}\left(X_{1}^{n}\right) & \geq E\;H(X_{1}^{n}). \end{array}$$

The length of the PPM code or the PPM probability, respectively, have two notable properties. First, the PPM probability is a universal probability, i.e., in the limit, the length of the PPM code consistently estimates the entropy rate of a stationary source. Second, the PPM probability can be effectively computed, i.e., the summation in definition (40) can be rewritten as a finite sum. Let us state these two results formally.

**Theorem** **6** (cf. [39])**.** *The PPM probability is universal in expectation, i.e., we have*

(43) $$\begin{array}{cc} \lim_{n\to \infty }\frac{1}{n}E\;H_{\mathrm{PPM}}\left(X_{1}^{n}\right) & =\lim_{n\to \infty }\frac{1}{n}E\;H\left(X_{1}^{n}\right) \end{array}$$

*for any stationary process ${\left(X_{i}\right)}_{i=1}^{\infty }$.*

For stationary ergodic processes, the above claim follows by an iterated application of the ergodic theorem as shown, e.g., in Theorem 1.1 from [39] for the measure *R*, which is a slight modification of the PPM probability. To generalize the claim for nonergodic processes, one can use the ergodic decomposition theorem, but the exact proof requires too large of a theoretical overload to be presented within the framework of this paper.

**Theorem** **7.** *The PPM probability can be effectively computed, i.e., we have*

(44) $$\begin{array}{c} \mathrm{PPM}\left(x_{1}^{n}\right)=\frac{6}{\pi^{2}}\sum_{k=0}^{L(x_{1}^{n})}\frac{{\mathrm{PPM}}_{k}\left(x_{1}^{n}\right)}{{\left(k+2\right)}^{2}}+\left(1-\frac{6}{\pi^{2}}\sum_{k=0}^{L(x_{1}^{n})}\frac{1}{{\left(k+2\right)}^{2}}\right)D^{-n}, \end{array}$$

*where*

(45) $$\begin{array}{c} L\left(x_{1}^{n}\right)=\max\left\{k:N(w_{1}^{k}|x_{1}^{n})>1\;\mathrm{for}\;\mathrm{some}\;w_{1}^{k}\right\} \end{array}$$

*is the maximal repetition of string $x_{1}^{n}$.*

**Proof.**  We have $N(x_{i-k}^{i-1}|x_{1}^{i-2})=0$ for $k>L(x_{1}^{i})$. Hence, ${\mathrm{PPM}}_{k}\left(x_{1}^{n}\right)=D^{-n}$ for $k>L(x_{1}^{n})$ and, in view of this, we obtain the claim. ☐

Maximal repetition as a function of a string was studied, e.g., in [40,41]. Since the PPM probability is a computable probability distribution, then, by (27) for a certain constant $C_{\mathrm{PPM}},$ we have

(46) $$\begin{array}{c} H_{a}\left(X_{1}^{n}\right)\leq H_{\mathrm{PPM}}\left(X_{1}^{n}\right)+2\log n+C_{\mathrm{PPM}}. \end{array}$$

Let us denote the length of the PPM code of order *k*,

(47) $$\begin{array}{cc} H_{{\mathrm{PPM}}_{k}}\left(x_{1}^{n}\right) & :=-\log{\mathrm{PPM}}_{k}\left(x_{1}^{n}\right). \end{array}$$

As we can easily see, the code length $H_{\mathrm{PPM}}\left(x_{1}^{n}\right)$ is approximately equal to the minimal code length $H_{{\mathrm{PPM}}_{k}}\left(x_{1}^{n}\right)$ where the minimization goes over $k\in \left\{-1,0,1,\ldots \right\}$. Thus, it is meaningful to consider this definition of the PPM order of an arbitrary string.

**Definition** **4.** *The* PPM order *$G_{\mathrm{PPM}}\left(x_{1}^{n}\right)$ is the smallest G such that*

(48) $$\begin{array}{c} H_{{\mathrm{PPM}}_{G}}\left(x_{1}^{n}\right)\leq H_{{\mathrm{PPM}}_{k}}\left(x_{1}^{n}\right)\;\mathrm{for}\;\mathrm{all}\;k\geq -1. \end{array}$$

**Theorem** **8.** We have $G_{\mathrm{PPM}}\left(x_{1}^{n}\right)\leq L\left(x_{1}^{n}\right)$.

**Proof.**  It follows by ${\mathrm{PPM}}_{k}\left(x_{1}^{n}\right)=D^{-n}={\mathrm{PPM}}_{-1}\left(x_{1}^{n}\right)$ for $k>L(x_{1}^{n})$. ☐

Let us divert for a short while from the PPM code definition. The set of distinct substrings of length *m* in string $x_{1}^{n}$ is

(49) $$\begin{array}{c} V\left(m|x_{1}^{n}\right):=\left\{y_{1}^{m}:x_{t+1}^{t+m}=y_{1}^{m}\;\mathrm{for}\;\mathrm{some}\;0\leq t\leq n-m\right\}. \end{array}$$

The cardinality of set $V(m|x_{1}^{n})$ as a function of substring length *m* is called the subword complexity of string $x_{1}^{n}$ [40]. Now let us apply the concept of the PPM order to define some special set of substrings of an arbitrary string $x_{1}^{n}$. The set of distinct PPM words detected in $x_{1}^{n}$ will be defined as the set $V(m|x_{1}^{n})$ for $m=G_{\mathrm{PPM}}\left(x_{1}^{n}\right)$, i.e.,

(50) $$\begin{array}{c} V_{\mathrm{PPM}}\left(x_{1}^{n}\right):=V\left(G_{\mathrm{PPM}}\left(X_{1}^{n}\right)|x_{1}^{n}\right). \end{array}$$

Let us define the pointwise mutual information

(51) $$\begin{array}{c} I(X;Y):=H(X)+H(Y)-H(X,Y) \end{array}$$

and the algorithmic mutual information

(52) $$\begin{array}{c} I_{a}\left(u;v\right):=H_{a}\left(u\right)+H_{a}\left(v\right)-H_{a}\left(u,v\right). \end{array}$$

Now, we may write down the theorem about facts and words. The theorem states that the Hilberg exponent for the expected number of initial independent inferrable facts is less than the Hilberg exponent for the expected mutual information and this is less than the Hilberg exponent for the expected number of distinct detected PPM words plus the PPM order. (The PPM order is usually much less than the number of distinct PPM words.)

**Theorem** **9** (facts and words I, cf. [25])**.** *Let ${\left(X_{i}\right)}_{i=1}^{\infty }$ be a stationary strongly nonergodic process over a finite alphabet. We have inequalities*

(53) $$\begin{array}{cc} \underset{n\to \infty }{\mathrm{hilb}}E\;\mathrm{card}U\left(X_{1}^{n}\right) & \leq \underset{n\to \infty }{\mathrm{hilb}}E\;I\left(X_{1}^{n};X_{n+1}^{2n}\right)\\ & \leq \underset{n\to \infty }{\mathrm{hilb}}E\;\left[G_{\mathrm{PPM}}\left(X_{1}^{n}\right)+\mathrm{card}V_{\mathrm{PPM}}\left(X_{1}^{n}\right)\right]. \end{array}$$

**Proof.**  The claim follows by conjunction of Theorem A2 from Appendix A and Theorem A8 from Appendix B. ☐

Theorem 9 also has an algorithmic version, for ergodic processes in particular.

**Theorem** **10** (facts and words II)**.** *Let ${\left(X_{i}\right)}_{i=1}^{\infty }$ be a stationary process over a finite alphabet. We have inequalities*

(54) $$\begin{array}{cc} \underset{n\to \infty }{\mathrm{hilb}}E\;\mathrm{card}U_{a}\left(X_{1}^{n}\right) & \leq \underset{n\to \infty }{\mathrm{hilb}}E\;I_{a}\left(X_{1}^{n};X_{n+1}^{2n}\right)\\ & \leq \underset{n\to \infty }{\mathrm{hilb}}E\;\left[G_{\mathrm{PPM}}\left(X_{1}^{n}\right)+\mathrm{card}V_{\mathrm{PPM}}\left(X_{1}^{n}\right)\right]. \end{array}$$

**Proof.**  The claim follows by conjunction of Theorem A3 from Appendix A and Theorem A8 from Appendix B. ☐

The theorem about facts and words previously proven in [25] differs from Theorem 9 in three aspects. First of all, the theorem in [25] did not apply the concept of the Hilberg exponent and compared ${\lim\;\inf}_{n\to \infty }$ with ${\lim\;\sup}_{n\to \infty }$ rather than ${\lim\;\sup}_{n\to \infty }$ with ${\lim\;\sup}_{n\to \infty }$. Second, the number of inferrable facts was defined as a functional of the process distribution rather than a random variable depending on a particular text. Third, the number of words was defined using a minimal grammar-based code rather than the concept of the PPM order. Minimal grammar-based codes are not computable in a polynomial time in contrast to the PPM order. Thus, we may claim that Theorem 9 is stronger than the theorem about facts and words previously proven in [25]. Moreover, applying Kolmogorov complexity and algorithmic randomness to formulate and prove Theorem 10 is a new idea.

It is an interesting question whether we have an almost sure version of Theorems 9 and 10, namely, whether

(55) $$\begin{array}{cc} {\mathrm{hilb}}_{n\to \infty }\mathrm{card}U\left(X_{1}^{n}\right) & \leq {\mathrm{hilb}}_{n\to \infty }I\left(X_{1}^{n};X_{n+1}^{2n}\right)\\ & \leq {\mathrm{hilb}}_{n\to \infty }\left[G_{\mathrm{PPM}}\left(X_{1}^{n}\right)+\mathrm{card}V_{\mathrm{PPM}}\left(X_{1}^{n}\right)\right]\;\mathrm{almost}\;\mathrm{surely} \end{array}$$

for strongly nonergodic processes, or

(56) $$\begin{array}{cc} {\mathrm{hilb}}_{n\to \infty }\mathrm{card}U_{a}\left(X_{1}^{n}\right) & \leq {\mathrm{hilb}}_{n\to \infty }I_{a}\left(X_{1}^{n};X_{n+1}^{2n}\right)\\ & \leq {\mathrm{hilb}}_{n\to \infty }\left[G_{\mathrm{PPM}}\left(X_{1}^{n}\right)+\mathrm{card}V_{\mathrm{PPM}}\left(X_{1}^{n}\right)\right]\;\mathrm{almost}\;\mathrm{surely} \end{array}$$

for general stationary processes. We leave this question as an open problem.

## 6. Hilberg Exponents and Empirical Data

It is advisable to show that the Hilberg exponents considered in Theorem 9 can assume any value in range [0,1] and the difference between them can be arbitrarily large. We adopt a convention that the set of inferrable probabilistic facts is empty for ergodic processes, $U(X_{1}^{n})=\emptyset$. With this remark in mind, let us inspect some examples of processes.

First of all, for Markov processes and their strongly nonergodic mixtures, of any order *k*, but, over a finite alphabet, we have

(57) $$\begin{array}{c} \underset{n\to \infty }{\mathrm{hilb}}E\;\mathrm{card}U\left(X_{1}^{n}\right)=\underset{n\to \infty }{\mathrm{hilb}}E\;I\left(X_{1}^{n};X_{n+1}^{2n}\right)=0. \end{array}$$

This happens to be so since the sufficient statistic of text $X_{1}^{n}$ for predicting text $X_{n+1}^{2n}$ is the maximum likelihood estimate of the transition matrix, the elements of which can assume at most (n+1) distinct values. Hence, $E\;I\left(X_{1}^{n};X_{n+1}^{2n}\right)\leq D^{k+1}\log\left(n+1\right)$, where *D* is the cardinality of the alphabet and *k* is the Markov order of the process. Similarly, it can be shown for these processes that the PPM order satisfies $\lim_{n\to \infty }G_{\mathrm{PPM}}\left(X_{1}^{n}\right)\leq k$. Hence, the number of PPM words, which satisfies inequality $\mathrm{card}V_{\mathrm{PPM}}\left(X_{1}^{n}\right)\leq D^{G_{\mathrm{PPM}}\left(X_{1}^{n}\right)}$, is also bounded above. In consequence, for Markov processes and their strongly nonergodic mixtures, of any order but over a finite alphabet, we obtain

(58) $$\begin{array}{c} \underset{n\to \infty }{\mathrm{hilb}}\left[G_{\mathrm{PPM}}\left(X_{1}^{n}\right)+\mathrm{card}V_{\mathrm{PPM}}\left(X_{1}^{n}\right)\right]=0\;\mathrm{almost}\;\mathrm{surely}. \end{array}$$

In contrast, Santa Fe processes are strongly nonergodic mixtures of some IID processes over an infinite alphabet. Being mixtures of IID processes over an infinite alphabet, they need not satisfy condition (58). In fact, as shown in [25,29] and Appendix C, for the Santa Fe process with exponent α, we have the asymptotic power-law growth

(59) $$\begin{array}{c} \underset{n\to \infty }{\mathrm{hilb}}E\;\mathrm{card}U\left(X_{1}^{n}\right)=\underset{n\to \infty }{\mathrm{hilb}}E\;I\left(X_{1}^{n};X_{n+1}^{2n}\right)=1/\alpha \in \left(0,1\right). \end{array}$$

The same equality for the number of inferrable probabilistic facts and the mutual information is also satisfied by a stationary coding of the Santa Fe process into a finite alphabet (see [29]).

Let us also note that, whereas the theorem about facts and words provides an inequality of Hilberg exponents, this inequality can be strict. To provide some substance, in [29], we have constructed a modification of the Santa Fe process that is ergodic and over a finite alphabet. For this modification, we have only the power-law growth of mutual information

(60) $$\begin{array}{cc} \underset{n\to \infty }{\mathrm{hilb}}E\;I\left(X_{1}^{n};X_{n+1}^{2n}\right) & =1/\alpha \in (0,1). \end{array}$$

Since, in this case, ${\mathrm{hilb}}_{n\to \infty }E\;\mathrm{card}U\left(X_{1}^{n}\right)=0$, then the difference between the Hilberg exponents for the number of inferrable probabilistic facts and the number of PPM words can be an arbitrary number in range (0,1).

**Figure 1:**
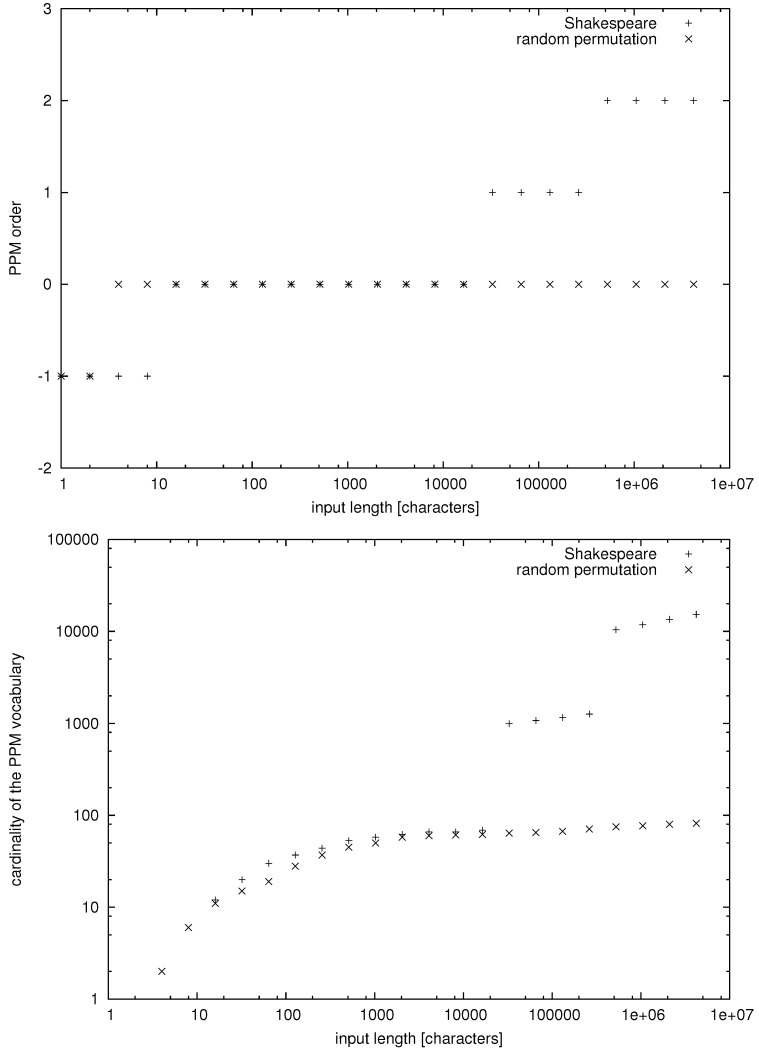
The PPM order $G_{\mathrm{PPM}}\left(x_{1}^{n}\right)$ and the cardinality of the PPM vocabulary $\mathrm{card}V_{\mathrm{PPM}}\left(x_{1}^{n}\right)$ versus the input length *n* for William Shakespeare’s First Folio/35 Plays and a random permutation of the text’s characters.

Now, we are in a position to discuss some empirical data. In this case, we cannot directly measure the number of facts and the mutual information, but we can compute the PPM order and count the number of PPM words. In Figure 1, we have presented data for a collection of 35 plays by William Shakespeare (downloaded from the Project Gutenberg, https://www.gutenberg.org/) and a random permutation of characters appearing in this collection of texts. The random permutation of characters is an IID process over a finite alphabet, so, in this case, we obtain

(61) $$\begin{array}{c} \underset{n\to \infty }{\mathrm{hilb}}\mathrm{card}V_{\mathrm{PPM}}\left(x_{1}^{n}\right)=0. \end{array}$$

In contrast, for the plays of Shakespeare, we seem to have a stepwise power law growth of the number of distinct PPM words. Thus, we may suppose that, for natural language, we have more generally

(62) $$\begin{array}{c} \underset{n\to \infty }{\mathrm{hilb}}\mathrm{card}V_{\mathrm{PPM}}\left(x_{1}^{n}\right)>0. \end{array}$$

If relationship (62) holds true, then natural language cannot be a Markov process of any order. Moreover, in view of the striking difference between observations (61) and (62), we may suppose that the number of inferrable probabilistic or algorithmic facts for texts in natural language also obeys a power-law growth. Formally speaking, this condition would translate to natural language being strongly nonergodic or perigraphic. We note that this hypothesis arises only as a form of a weak inductive inference since formally we cannot deduce condition (33) from mere condition (62), regardless of the amount of data supporting condition (62).

## 7. Conclusions

In this article, a stationary process has been called strongly nonergodic if some persistent random topic can be detected in the process and an infinite number of independent binary random variables, called probabilistic facts, is needed to describe this topic completely. Replacing probabilistic facts with an algorithmically random sequence of bits, called algorithmic facts, we have adapted this property back to ergodic processes. Subsequently, we have called a process perigraphic if the number of algorithmic facts which can be inferred from a finite text sampled from the process grows like a power of the text length.

We have demonstrated an assertion, which we call the theorem about facts and words. This proposition states that the number of independent probabilistic or algorithmic facts which can be inferred from a text drawn from a process must be roughly smaller than the number of distinct word-like strings detected in this text by means of the PPM compression algorithm. We have exhibited two versions of this theorem: one for strongly nonergodic processes, applying the Shannon information theory, and one for ergodic processes, applying the algorithmic information theory.

Subsequently, we have exhibited an empirical observation that the number of distinct word-like strings grows like a stepwise power law for a collection of plays by William Shakespeare, in stark contrast to Markov processes. This observation does not rule out that the number of probabilistic or algorithmic facts inferrable from texts in natural language also grows like a power law. Hence, we have supposed that natural language is a perigraphic process.

We suppose that the path of the future related research should lead through a further analysis of the theorem about facts and words, and demonstrating an almost sure version of this statement. It is also an important, still unresolved question whether theoretical analysis of effective universal coding algorithms and their rates of convergence to the entropy rate can contribute to some definite statements about natural language treated as a stochastic process. We realize that the results of this paper as far as the linguistic theory is concerned may be still too inconclusive. As we see it, the main merit of this paper lies in linking some concepts in the Shannon information theory and the algorithmic information theory and providing some linguistic interpretations of them.

## Abbreviations

The following abbreviations are used in this manuscript:

IID	independent identically distributed
PPM	prediction by partial matching

## Appendix A. Facts and Mutual Information

In the appendices, we will make use of several kinds of information measures.

1. First, there are four pointwise Shannon information measures:
  - entropy
    H(X)=−logP(X),
  - conditional entropy
    H(X|Z):=−logP(X|Z),
  - mutual information
    I(X;Y):=H(X)+H(Y)−H(X,Y),
  - conditional mutual information
    I(X;Y|Z):=H(X|Z)+H(Y|Z)−H(X,Y|Z),
  where P(X) is the probability of a random variable *X* and P(X|Z) is the conditional probability of a random variable *X* given a random variable *Z*. The above definitions make sense for discrete-valued random variables *X* and *Y* and an arbitrary random variable *Z*. If *Z* is a discrete-valued random variable, then also H(X,Z)−H(Z)=H(X|Z) and I(X;Z)=H(X)−H(X|Z).
2. Moreover, we will use four algorithmic information measures:
  - entropy
    $H_{a}\left(x\right)=K\left(x\right)\log2$,
  - conditional entropy
    $H_{a}\left(x|z\right):=K\left(x|z\right)\log2$,
  - mutual information
    $I_{a}\left(x;y\right):=H_{a}\left(x\right)+H_{a}\left(y\right)-H_{a}\left(x,y\right)$,
  - conditional mutual information
    $I_{a}\left(x;y|z\right):=H_{a}\left(x|z\right)+H_{a}\left(y|z\right)-H_{a}\left(x,y|z\right)$,
  where K(x) is the prefix-free Kolmogorov complexity of an object *x* and K(x|z) is the prefix-free Kolmogorov complexity of an object *x* given an object *z*. In the above definitions, *x* and *y* must be finite objects (finite texts), whereas *z* can be also an infinite object (an infinite sequence). If *z* is a finite object, then $H_{a}\left(x,z\right)-H_{a}\left(z\right)\overset{+}{=}H_{a}\left(x|z,K\left(z\right)\right)$ rather than being equal to $H_{a}\left(x|z\right)$, where $\overset{+}{=}$, $\overset{+}{<}$, and $\overset{+}{>}$ are the equality and the inequalities up to an additive constant ([37], Theorem 3.9.1). Hence,
  (A1) $$\begin{array}{cc} H_{a}\left(x\right)-H_{a}\left(x|z\right)+H_{a}\left(K\left(z\right)\right) & \overset{+}{>}I_{a}\left(x;z\right)\overset{+}{=}H_{a}\left(x\right)-H_{a}\left(x|z,K\left(z\right)\right)\\ & \overset{+}{>}H_{a}\left(x\right)-H_{a}\left(x|z\right). \end{array}$$

In the following, we will prove a result for Hilberg exponents.

**Theorem** **A1.** *For a function G:N→R, define J(n):=2G(n)−G(2n). If the limit $\lim_{n\to \infty }G\left(n\right)/n=g$ exists and is finite, then*

(A2) $$\begin{array}{c} \underset{n\to \infty }{\mathrm{hilb}}\left[G(n)-ng\right]\leq \underset{n\to \infty }{\mathrm{hilb}}J\left(n\right), \end{array}$$

*with an equality if $J\left(2^{n}\right)\overset{+}{>}0$ for all but finitely many n.*

**Proof.** The proof makes use of the telescope sum

(A3) $$\begin{array}{c} \sum_{k=0}^{\infty }\frac{J(2^{k+n})}{2^{k+1}}=G\left(2^{n}\right)-2^{n}g. \end{array}$$

Denote $\delta :={\mathrm{hilb}}_{n\to \infty }J\left(n\right)$. Since ${\mathrm{hilb}}_{n\to \infty }\left(G(n)-ng\right)\leq 1$, it is sufficient to prove inequality (A2) for δ<1. In this case, $J\left(2^{n}\right)\leq 2^{(\delta +\epsilon )n}$ for all but finitely many *n* for any ϵ>0. Then, for ϵ<1−δ, by the telescope sum (A3), we obtain for sufficiently large *n* that

(A4) $$\begin{array}{c} G\left(2^{n}\right)-2^{n}g\leq \sum_{k=0}^{\infty }\frac{2^{\left(\delta +\epsilon )(k+n\right)}}{2^{k+1}}\leq 2^{(\delta +\epsilon )n}\sum_{k=0}^{\infty }2^{(\delta +\epsilon -1)k-1}=\frac{2^{(\delta +\epsilon )n}}{2(1-2^{\delta +\epsilon -1})}. \end{array}$$

Since ϵ can be taken arbitrarily small, we obtain (A2). Now assume that $J\left(2^{n}\right)\overset{+}{>}0$ for all but finitely many *n*. By the telescope sum (A3), we have $J\left(2^{n}\right)/2\overset{+}{<}G\left(2^{n}\right)-2^{n}g$ for sufficiently large *n*. Hence,

(A5) $$\begin{array}{c} \delta \leq \underset{n\to \infty }{\mathrm{hilb}}\left(G(n)-ng\right) \end{array}$$

Combining this with (A2), we obtain ${\mathrm{hilb}}_{n\to \infty }\left(G(n)-ng\right)=\delta$. ☐

For any stationary process ${\left(X_{i}\right)}_{i=1}^{\infty }$ over a finite alphabet, there exists a limit

(A6) $$\begin{array}{cc} h & :=\lim_{n\to \infty }\frac{E\;H(X_{1}^{n})}{n}=E\;H\left(X_{1}|X_{2}^{\infty }\right), \end{array}$$

called the entropy rate of process ${\left(X_{i}\right)}_{i=1}^{\infty }$ [3]. By (28), (43), and (46), we also have

(A7) $$\begin{array}{cc} h & =\lim_{n\to \infty }\frac{E\;H_{a}\left(X_{1}^{n}\right)}{n}. \end{array}$$

Moreover, for a stationary process, the mutual information satisfies

(A8) $$\begin{array}{cc} E\;I(X_{1}^{n};X_{n+1}^{2n}) & =2E\;H\left(X_{1}^{n}\right)-E\;H\left(X_{1}^{2n}\right)\geq 0, \end{array}$$

(A9) $$\begin{array}{cc} E\;I_{a}\left(X_{1}^{n};X_{n+1}^{2n}\right) & =2E\;H_{a}\left(X_{1}^{n}\right)-E\;H_{a}\left(X_{1}^{2n}\right)\overset{+}{>}0. \end{array}$$

Hence, by Theorem A1, we obtain

(A10) $$\begin{array}{cc} \underset{n\to \infty }{\mathrm{hilb}}\left[E\;H(X_{1}^{n})-hn\right] & =\underset{n\to \infty }{\mathrm{hilb}}E\;I\left(X_{1}^{n};X_{n+1}^{2n}\right), \end{array}$$

(A13) $$\begin{array}{cc} \underset{n\to \infty }{\mathrm{hilb}}\left[E\;H_{a}\left(X_{1}^{n}\right)-hn\right] & =\underset{n\to \infty }{\mathrm{hilb}}E\;I_{a}\left(X_{1}^{n};X_{n+1}^{2n}\right). \end{array}$$

Subsequently, we will prove the initial parts of Theorems 9 and 10, i.e., the two versions of the theorem about facts and words. The probabilistic statement for strongly nonergodic processes goes first.

**Theorem** **A2** (facts and mutual information I)**.** *Let ${\left(X_{i}\right)}_{i=1}^{\infty }$ be a stationary strongly nonergodic process over a finite alphabet. We have inequality*

(A12) $$\begin{array}{cc} \underset{n\to \infty }{\mathrm{hilb}}E\;\mathrm{card}U\left(X_{1}^{n}\right) & \leq \underset{n\to \infty }{\mathrm{hilb}}E\;I\left(X_{1}^{n};X_{n+1}^{2n}\right). \end{array}$$

**Proof.** Let us write $S_{n}:=\mathrm{card}U\left(X_{1}^{n}\right)$. Observe that

(A13) $$\begin{array}{cc} E\;H(Z_{1}^{S_{n}}|S_{n}) & =-\sum_{s,w}P\left(S_{n}=s,Z_{1}^{s}=w\right)\log P\left(Z_{1}^{s}=w|S_{n}=s\right)\\ & \geq -\sum_{s,w}P\left(S_{n}=s,Z_{1}^{s}=w\right)\log\frac{P(Z_{1}^{s}=w)}{P(S_{n}=s)}\\ & =-\sum_{s,w}P\left(S_{n}=s,Z_{1}^{s}=w\right)\log\frac{2^{-s}}{P(S_{n}=s)}\\ & =\left(\log2\right)E\;S_{n}-E\;H\left(S_{n}\right), \end{array}$$

(A14) $$\begin{array}{cc} E\;H(S_{n}) & \leq \left(E\;S_{n}+1\right)\log\left(E\;S_{n}+1\right)-E\;S_{n}\log E\;S_{n}\\ & =\log\left(E\;S_{n}+1\right)+E\;S_{n}\log\frac{E\;S_{n}+1}{E\;S_{n}}\\ & \leq \log(E\;S_{n}+1)+1, \end{array}$$

where the second row of inequalities follows by the maximum entropy bound from ([3], Lemma 13.5.4). Hence, by the inequality

(A15) $$\begin{array}{c} E\;H(X|Y)\leq E\;H(X|f(Y)) \end{array}$$

for a measurable function *f*, we obtain that

(A16) $$\begin{array}{cc} E\;H\left(X_{1}^{n}\right)-E\;H\left(X_{1}^{n}|Z_{1}^{\infty }\right) & \geq E\;H\left(X_{1}^{n}|S_{n}\right)-E\;H\left(X_{1}^{n}|Z_{1}^{\infty },S_{n}\right)-E\;H\left(S_{n}\right)\\ & \geq E\;H\left(X_{1}^{n}|S_{n}\right)-E\;H\left(X_{1}^{n}|Z_{1}^{S_{n}},S_{n}\right)-E\;H\left(S_{n}\right)\\ & =E\;I\left(X_{1}^{n};Z_{1}^{S_{n}}|S_{n}\right)-E\;H\left(S_{n}\right)\\ & \geq E\;H\left(Z_{1}^{S_{n}}|S_{n}\right)-E\;H\left(Z_{1}^{S_{n}}|X_{1}^{n},S_{n}\right)-E\;H\left(S_{n}\right)\\ & =E\;H\left(Z_{1}^{S_{n}}|S_{n}\right)-E\;H\left(S_{n}\right)\\ & \geq \left(\log2\right)E\;S_{n}-2E\;H\left(S_{n}\right)\\ & \geq \left(\log2\right)E\;S_{n}-2\left[\log(E\;S_{n}+1)+1\right]. \end{array}$$

Now, we observe that

(A17) $$\begin{array}{c} E\;H\left(X_{1}^{n}|Z_{1}^{\infty }\right)\geq E\;H\left(X_{1}^{n}|X_{n+1}^{\infty }\right)=hn \end{array}$$

since the sequence of random variables $Z_{1}^{\infty }$ is a measurable function of the sequence of random variables $X_{n+1}^{\infty }$, as shown in [24,25]. Hence, we have

(A18) $$\begin{array}{c} E\;H\left(X_{1}^{n}\right)-E\;H\left(X_{1}^{n}|Z_{1}^{\infty }\right)\leq E\;H\left(X_{1}^{n}\right)-hn. \end{array}$$

By inequalities (A17) and (A18) and equality (A10), we obtain inequality (A12). ☐

The algorithmic version of the theorem about facts and words follows roughly the same idea, with some necessary adjustments.

**Theorem** **A3** (facts and mutual information II)**.** *Let ${\left(X_{i}\right)}_{i=1}^{\infty }$ be a stationary process over a finite alphabet. We have inequality*

(A19) $$\begin{array}{cc} \underset{n\to \infty }{\mathrm{hilb}}E\;\mathrm{card}U_{a}\left(X_{1}^{n}\right) & \leq \underset{n\to \infty }{\mathrm{hilb}}E\;I_{a}\left(X_{1}^{n};X_{n+1}^{2n}\right). \end{array}$$

**Proof.** Let us write $S_{n}:=\mathrm{card}U_{a}\left(X_{1}^{n}\right)$. Observe that

(A20) $$\begin{array}{cc} H_{a}\left(z_{1}^{S_{n}}|S_{n}\right) & \overset{+}{>}H_{a}\left(z_{1}^{S_{n}}\right)-H_{a}\left(S_{n}\right)\\ & \overset{+}{=}\left(\log2\right)S_{n}-C-H_{a}\left(S_{n}\right), \end{array}$$

(A21) $$\begin{array}{cc} H_{a}\left(S_{n}\right) & \overset{+}{<}2\log\left(S_{n}+1\right), \end{array}$$

(A22) $$\begin{array}{cc} H_{a}\left(K\left(z_{1}^{S_{n}}\right)\right) & \overset{+}{<}2\log\left(K\left(z_{1}^{S_{n}}\right)+1\right)\\ & \overset{+}{<}2\log\left(S_{n}+1\right), \end{array}$$

where the first row of inequalities follows by the algorithmic randomness of $z_{1}^{\infty }$, whereas the second and the third row of inequalities follow by the bounds $K\left(n\right)\overset{+}{<}2\log_{2}\left(n+1\right)$ for n≥0 and $K\left(z_{1}^{k}\right)\overset{+}{<}2k$. Moreover, for any a computable function *f*, there exists a constant $C_{f}\geq 0$ such that

(A23) $$\begin{array}{c} H_{a}\left(x|y\right)\overset{+}{<}H_{a}\left(x|f\left(y\right)\right)+C_{f}. \end{array}$$

Hence, we obtain that

(A24) $$\begin{array}{cc} H_{a}\left(X_{1}^{n}\right)-H_{a}\left(X_{1}^{n}|z_{1}^{\infty }\right) & \overset{+}{>}H_{a}\left(X_{1}^{n}|S_{n}\right)-H_{a}\left(X_{1}^{n}|z_{1}^{\infty },S_{n}\right)-H_{a}\left(S_{n}\right)\\ & \overset{+}{>}H_{a}\left(X_{1}^{n}|S_{n}\right)-H_{a}\left(X_{1}^{n}|z_{1}^{S_{n}},S_{n}\right)-H_{a}\left(S_{n}\right)\\ & \overset{+}{>}I_{a}\left(X_{1}^{n};z_{1}^{S_{n}}|S_{n}\right)-H_{a}\left(K\left(z_{1}^{S_{n}}\right)\right)-H_{a}\left(S_{n}\right)\\ & \overset{+}{>}H_{a}\left(z_{1}^{S_{n}}|S_{n}\right)-H_{a}\left(z_{1}^{S_{n}}|X_{1}^{n},K\left(X_{1}^{n}\right),S_{n}\right)\\ & \;-H_{a}\left(K\left(z_{1}^{S_{n}}\right)\right)-H_{a}\left(S_{n}\right)\\ & \overset{+}{>}H_{a}\left(z_{1}^{S_{n}}|S_{n}\right)-C_{g}-H_{a}\left(K\left(z_{1}^{S_{n}}\right)\right)-H_{a}\left(S_{n}\right)\\ & \overset{+}{>}\left(\log2\right)S_{n}-C-C_{g}-H_{a}\left(K\left(z_{1}^{S_{n}}\right)\right)-2H_{a}\left(S_{n}\right)\\ & \overset{+}{>}\left(\log2\right)S_{n}-6\log\left(S_{n}+1\right)-C-C_{g}. \end{array}$$

Since $-E\;\log\left(S_{n}+1\right)\geq -\log\left(E\;S_{n}+1\right)$ by the Jensen inequality, then

(A25) $$\begin{array}{c} E\;H_{a}\left(X_{1}^{n}\right)-E\;H_{a}\left(X_{1}^{n}|z_{1}^{\infty }\right)\overset{+}{>}\left(\log2\right)E\;S_{n}-6\log\left(E\;S_{n}+1\right)-C-C_{g}. \end{array}$$

Now, we observe that

(A26) $$\begin{array}{c} E\;H_{a}\left(X_{1}^{n}|z_{1}^{\infty }\right)\geq E\;H\left(X_{1}^{n}\right)\geq hn \end{array}$$

since the conditional prefix-free Kolmogorov complexity with the second argument fixed is the length of a prefix-free code. Hence, we have

(A27) $$\begin{array}{c} E\;H_{a}\left(X_{1}^{n}\right)-E\;H_{a}\left(X_{1}^{n}|z_{1}^{\infty }\right)\leq E\;H_{a}\left(X_{1}^{n}\right)-hn. \end{array}$$

By inequalities (A25) and (A27) and equality (A11), we obtain inequality (A19). ☐

## Appendix B. Mutual Information and PPM Words

In this appendix, we will investigate some algebraic properties of the length of the PPM code to be used for proving the second part of the theorem about facts and words. First of all, it can be seen that

(A28) $$\begin{array}{c} H_{{\mathrm{PPM}}_{k}}\left(x_{1}^{n}\right)=\left\{\begin{array}{cc} n\log D, & k=-1,\\ k\log D+\sum_{u\in X^{k}}\log\frac{(N\left(u|x_{1}^{n-1}\right)+D-1)!}{\left(D-1\right)!\prod_{a=1}^{D}N\left(ua|x_{1}^{n}\right)!}, & k\geq 0. \end{array}\right. \end{array}$$

Expression (A28) can be further rewritten using notation

(A29) $$\begin{array}{cc} \log^{*}n & :=\left\{\begin{array}{cc} 0, & n=0,\\ \log n!-n\log n+n, & n\geq 1, \end{array}\right. \end{array}$$

(A30) $$\begin{array}{cc} H(n_{1},\ldots ,n_{l}) & :=\left\{\begin{array}{cc} \sum_{i=1:n_{i}>0}^{l}n_{i}\log\left(\frac{\sum_{j=1}^{l}n_{j}}{n_{i}}\right), & \mathrm{if}\;n_{j}>0\;\mathrm{exists},\\ 0, & \mathrm{else}, \end{array}\right. \end{array}$$

(A31) $$\begin{array}{cc} K(n_{1},\ldots ,n_{l}) & :=\sum_{i=1}^{l}\log^{*}n_{i}-\log^{*}\left(\sum_{i=1}^{l}n_{i}\right). \end{array}$$

Then, for k≥0, we define

(A32) $$\begin{array}{cc} H_{{\mathrm{PPM}}_{k}^{0}}\left(x_{1}^{n}\right) & :=\sum_{u\in X^{k}}H\left(N(u1|x_{1}^{n}),\ldots ,N(uD|x_{1}^{n})\right), \end{array}$$

(A33) $$\begin{array}{cc} H_{{\mathrm{PPM}}_{k}^{1}}\left(x_{1}^{n}\right): & =\sum_{u\in X^{k}}H\left(N(u|x_{1}^{n-1}),D-1\right)\\ & \;-\sum_{u\in X^{k}}K\left(N(u1|x_{1}^{n}),\ldots ,N(uD|x_{1}^{n}),D-1\right). \end{array}$$

As a result for k≥0, we obtain

(A34) $$\begin{array}{cc} H_{{\mathrm{PPM}}_{k}}\left(x_{1}^{n}\right) & =k\log D+H_{{\mathrm{PPM}}_{k}^{0}}\left(x_{1}^{n}\right)+H_{{\mathrm{PPM}}_{k}^{1}}\left(x_{1}^{n}\right). \end{array}$$

In the following, we will analyze the terms on the right-hand side of (A34).

**Theorem** **A4.** *For k≥0 and n≥1, we have*

(A35) $$\begin{array}{cc} \tilde{D}\mathrm{card}V\left(k|x_{1}^{n-1}\right) & \leq H_{{\mathrm{PPM}}_{k}^{1}}\left(x_{1}^{n}\right)<D\mathrm{card}V\left(k|x_{1}^{n-1}\right)\left(2+\log n\right), \end{array}$$

*where $\tilde{D}:=-D\log\left(D^{-1}\right)!>0$.*

**Proof.** Observe that H(0,D−1)=K(0,…,0,D−1)=0. Hence, the summation in $H_{{\mathrm{PPM}}_{k}^{1}}\left(x_{1}^{n}\right)$ can be restricted to $u\in X^{k}$ such that $N(u|x_{1}^{n-1})\geq 1$. Consider such a *u* and write $N=N(u|x_{1}^{n-1})$ and $N_{a}=N\left(ua|x_{1}^{n}\right)$. Since $H(n_{1},\ldots ,n_{l})\geq 0$ and $K(n_{1},\ldots ,n_{l})\geq 0$ (the second inequality follows by subadditivity of $\log^{*}n$), we obtain first

(A36) $$\begin{array}{cc} H\left(N,D-1\right)-K\left(N_{1},\ldots ,N_{D},D-1\right) & \leq H\left(N,D-1\right)\\ & =N\log\left(1+\frac{D-1}{N}\right)+\left(D-1\right)\log\left(1+\frac{N}{D-1}\right)\\ & \leq N\cdot \frac{D-1}{N}+\left(D-1\right)\log\left(1+\frac{N}{D-1}\right)\\ & =\left(D-1\right)\left[1+\log\left(1+\frac{N}{D-1}\right)\right]<D\left(2+\log n\right), \end{array}$$

where we use log(1+x)≤x and N<n. On the other hand, function $\log^{*}n$ is concave so by $\sum_{a=1}^{D}N_{a}=N$ and the Jensen inequality for $\log^{*}n$, we obtain

(A37) $$\begin{array}{c} H\left(N,D-1\right)-K\left(N_{1},\ldots ,N_{D},D-1\right)\geq F\left(N,D\right):\\ =N\log\left(1+\frac{D-1}{N}\right)+\left(D-1\right)\log\left(1+\frac{N}{D-1}\right)\\ \;+\log^{*}\left(N+D-1\right)-\log^{*}\left(D-1\right)-D\log^{*}\left(N/D\right)\\ =\log\left(N+D-1\right)!-\log\left(D-1\right)!-D\log\left(N/D\right)!-N\log D\\ =\log\frac{(N+D-1)!}{\left(D-1\right)!\left(N/D\right){!}^{D}D^{N}}\geq 0, \end{array}$$

since

(A38) $$\begin{array}{cc} \left(N/D\right){!}^{D}D^{N} & =N^{D}{\left(N-D\right)}^{D}{\left(N-2D\right)}^{D}\ldots D^{D}\\ & \leq \left(N+D-1\right)\left(N+D-2\right)\ldots D=\frac{(N+D-1)!}{(D-1)!}. \end{array}$$

Moreover, function $F\left(N,D\right)$ is growing in argument *N*. Hence,

(A39) $$\begin{array}{c} F\left(N,D\right)\geq F\left(1,D\right)=-D\log\left(D^{-1}\right)!. \end{array}$$

Summing inequalities (A36) and (A39) over $u\in X^{k}$ such that $N(u|x_{1}^{n})\geq 1$, we obtain the claim. ☐

The mutual information is defined as a difference of entropies. Replacing the entropy with an arbitrary function $H_{Q}\left(u\right)$, we obtain this quantity:

**Definition** **A1.** *The Q* pointwise mutual information *is defined as*

(A40) $$\begin{array}{c} I_{Q}\left(u;v\right):=H_{Q}\left(u\right)+H_{Q}\left(v\right)-H_{Q}\left(uv\right). \end{array}$$

We will show that the ${\mathrm{PPM}}_{k}^{0}$ pointwise mutual information cannot be positive.

**Theorem** **A5.** *For $n_{i}=\sum_{j=1}^{l}n_{ij}$, where $n_{ij}\geq 0$, we have*

(A41) $$\begin{array}{c} H\left(n_{1},\ldots ,n_{k}\right)\geq \sum_{j=1}^{l}H\left(n_{1j},\ldots ,n_{kj}\right). \end{array}$$

**Proof.** Write $N:=\sum_{i=1}^{k}\sum_{j=1}^{l}n_{ij}$, $p_{ij}:=n_{ij}/N$, $q_{i}:=\sum_{j=1}^{l}p_{ij}$, and $r_{j}:=\sum_{i=1}^{k}p_{ij}$. We observe that

(A42) $$\begin{array}{c} H\left(n_{1},\ldots ,n_{k}\right)-\sum_{j=1}^{l}H\left(n_{1j},\ldots ,n_{kj}\right)=N\sum_{i=1}^{k}\sum_{j=1}^{l}p_{ij}\log\frac{p_{ij}}{q_{i}r_{j}}, \end{array}$$

which is *N* times the Kullback–Leibler divergence between distributions $\left\{p_{ij}\right\}$ and $\left\{q_{i}r_{j}\right\}$ and thus is nonnegative. ☐

**Theorem** **A6.** *For k≥0, we have*

(A43) $$\begin{array}{c} I_{{\mathrm{PPM}}_{k}^{0}}\left(x_{1}^{n};x_{n+1}^{n+m}\right)\leq 0. \end{array}$$

**Proof.** Consider k≥0. For $u\in X^{k}$ and a∈X, we have

(A44) $$\begin{array}{cc} N(ua|x_{1}^{n+m}) & =N\left(ua|x_{1}^{n}\right)+N\left(ua|x_{n-k}^{n+k}\right)+N\left(ua|x_{n+1}^{n+m}\right). \end{array}$$

Thus, using Theorem A5, we obtain

(A45) $$\begin{array}{cc} H\left(N(u1|x_{1}^{n+m}),\ldots ,N(uD|x_{1}^{n+m})\right) & \geq H\left(N(u1|x_{1}^{n}),\ldots ,N(uD|x_{1}^{n})\right)\\ & +H\left(N(u1|x_{n-k}^{n+k}),\ldots ,N(uD|x_{n-k}^{n+k})\right)\\ & +H\left(N(u1|x_{n+1}^{n+m}),\ldots ,N(uD|x_{n+1}^{n+m})\right). \end{array}$$

Since the second term on the right-hand side is greater than or equal zero, we may omit it and summing the remaining terms over all $u\in X^{k}$ we obtain the claim. ☐

Now, we will show that the PPM pointwise mutual information between two parts of a string is roughly bounded above by the cardinality of the PPM vocabulary of the string multiplied by the logarithm of the string length.

**Theorem** **A7.** *We have*

(A46) $$\begin{array}{cc} I_{\mathrm{PPM}}\left(x_{1}^{n};x_{n+1}^{n+m}\right) & \leq 1+4\log\left[G_{\mathrm{PPM}}\left(x_{1}^{n+m}\right)+2\right]+\left[G_{\mathrm{PPM}}\left(x_{1}^{n+m}\right)+1\right]\log D\\ & \;+2D\mathrm{card}V_{\mathrm{PPM}}\left(x_{1}^{n+m}\right)\left[2+\log(n+m)\right]. \end{array}$$

**Proof.** Consider k≥0. By Theorems A4 and A6, we obtain

(A47) $$\begin{array}{cc} I_{{\mathrm{PPM}}_{k}}\left(x_{1}^{n};x_{n+1}^{n+m}\right) & =k\log D+I_{{\mathrm{PPM}}_{k}^{0}}\left(x_{1}^{n};x_{n+1}^{n+m}\right)+I_{{\mathrm{PPM}}_{k}^{1}}\left(x_{1}^{n};x_{n+1}^{n+m}\right)\\ & \leq k\log D+D\mathrm{card}V\left(k|x_{1}^{n}\right)\left[2+\log n\right]\\ & \;+D\mathrm{card}V\left(k|x_{n+1}^{n+m}\right)\left[2+\log m\right]\\ & \leq k\log D+2D\mathrm{card}V\left(k|x_{1}^{n+m}\right)\left[2+\log(n+m)\right]. \end{array}$$

In contrast, $I_{{\mathrm{PPM}}_{-1}}\left(x_{1}^{n};x_{n+1}^{n+m}\right)=0$. Now, let $G=G_{\mathrm{PPM}}\left(x_{1}^{n+m}\right)$. Since

(A48) $$\begin{array}{c} H_{\mathrm{PPM}}\left(x_{1}^{n+m}\right)\geq H_{{\mathrm{PPM}}_{G}}\left(x_{1}^{n+m}\right) \end{array}$$

and

(A49) $$\begin{array}{c} H_{\mathrm{PPM}}\left(u\right)\leq H_{{\mathrm{PPM}}_{k}}\left(u\right)+1/2+2\log\left(k+2\right) \end{array}$$

for any $u\in X^{*}$ and k≥−1, we obtain

(A50) $$\begin{array}{cc} I_{\mathrm{PPM}}\left(x_{1}^{n};x_{n+1}^{n+m}\right) & \leq I_{{\mathrm{PPM}}_{G}}\left(x_{1}^{n};x_{n+1}^{n+m}\right)+1+4\log\left(G+2\right)\\ & \leq 1+4\log\left(G+2\right)+\left(G+1\right)\log D\\ & \;+2D\mathrm{card}V\left(G|x_{1}^{n+m}\right)\left[2+\log(n+m)\right]. \end{array}$$

Hence, the claim follows. ☐

Consequently, we may prove the second part of Theorems 9 and 10, i.e., the theorems about facts and words.

**Theorem** **A8** (mutual information and words)**.** *Let ${\left(X_{i}\right)}_{i=1}^{\infty }$ be a stationary process over a finite alphabet. We have inequalities*

(A51) $$\begin{array}{cc} {\mathrm{hilb}}_{n\to \infty }E\;I\left(X_{1}^{n};X_{n+1}^{2n}\right) & \leq {\mathrm{hilb}}_{n\to \infty }E\;I_{a}\left(X_{1}^{n};X_{n+1}^{2n}\right)\\ & \leq {\mathrm{hilb}}_{n\to \infty }E\;\left[G_{\mathrm{PPM}}\left(X_{1}^{n}\right)+\mathrm{card}V_{\mathrm{PPM}}\left(X_{1}^{n}\right)\right]. \end{array}$$

**Proof.** By Theorem A7, we obtain

(A52) $$\begin{array}{cc} \underset{n\to \infty }{\mathrm{hilb}}E\;I_{\mathrm{PPM}}\left(X_{1}^{n};X_{n+1}^{2n}\right) & \leq \underset{n\to \infty }{\mathrm{hilb}}E\;\left[G_{\mathrm{PPM}}\left(X_{1}^{n}\right)+\mathrm{card}V_{\mathrm{PPM}}\left(X_{1}^{n}\right)\right]. \end{array}$$

In contrast, Theorems 6 and A1 and inequalities (28) and (46) yield

(A53) $$\begin{array}{cc} {\mathrm{hilb}}_{n\to \infty }\left[E\;H(X_{1}^{n})-hn\right] & \leq {\mathrm{hilb}}_{n\to \infty }\left[E\;H_{a}\left(X_{1}^{n}\right)-hn\right]\\ & \leq {\mathrm{hilb}}_{n\to \infty }\left[E\;H_{\mathrm{PPM}}\left(X_{1}^{n}\right)-hn\right]\\ & \leq {\mathrm{hilb}}_{n\to \infty }E\;I_{\mathrm{PPM}}\left(X_{1}^{n};X_{n+1}^{2n}\right). \end{array}$$

Hence, by equalities (A10) and (A11), we obtain inequality (A51). ☐

## Appendix C. Hilberg Exponents for Santa Fe Processes

We begin with a general observation for Hilberg exponents. In [36], this result was discussed only for the Hilberg exponent of mutual information.

**Theorem** **A9** (cf. [36]). *For a sequence of random variables $Y_{n}\geq 0$, we have*

(A54) $$\begin{array}{c} \underset{n\to \infty }{\mathrm{hilb}}Y_{n}\leq \underset{n\to \infty }{\mathrm{hilb}}E\;Y_{n}\;\mathrm{almost}\;\mathrm{surely}. \end{array}$$

**Proof.** Denote $\delta :={\mathrm{hilb}}_{n\to \infty }E\;Y_{n}$. From the Markov inequality, we have

(A55) $$\begin{array}{cc} \sum_{k=1}^{\infty }P\left(\frac{Y_{2^{k}}}{2^{k(\delta +\epsilon )}}\geq 1\right) & \leq \sum_{k=1}^{\infty }\frac{E\;Y_{2^{k}}}{2^{k(\delta +\epsilon )}}\\ & \leq A+\sum_{k=1}^{\infty }\frac{2^{k(\delta +\epsilon /2)}}{2^{k(\delta +\epsilon )}}<\infty , \end{array}$$

where A<∞. Hence, by the Borel–Cantelli lemma, we have $Y_{2^{k}}<2^{k(\delta +\epsilon )}$ for all but finitely many *n* almost surely. Since we can choose ϵ arbitrarily small, in particular, we obtain inequality (A54). ☐

In [29,36], it was shown that the Santa Fe process with exponent α satisfies equalities

(A56) $$\begin{array}{cc} \underset{n\to \infty }{\mathrm{hilb}}I\left(X_{-n+1}^{0};X_{1}^{n}\right) & =1/\alpha \;\mathrm{almost}\;\mathrm{surely}, \end{array}$$

(A57) $$\begin{array}{cc} \underset{n\to \infty }{\mathrm{hilb}}E\;I\left(X_{-n+1}^{0};X_{1}^{n}\right) & =1/\alpha . \end{array}$$

We will now show a similar result for the number of probabilistic facts inferrable from the Santa Fe process almost surely and in expectation. Since Santa Fe processes are processes over an infinite alphabet, we cannot apply the theorem about facts and words.

**Theorem** **A10.** *For the Santa Fe process with exponent α, we have*

(A58) $$\begin{array}{cc} \underset{n\to \infty }{\mathrm{hilb}}\mathrm{card}U\left(X_{1}^{n}\right) & =1/\alpha \;\mathrm{almost}\;\mathrm{surely}, \end{array}$$

(A59) $$\begin{array}{cc} \underset{n\to \infty }{\mathrm{hilb}}E\;\mathrm{card}U\left(X_{1}^{n}\right) & =1/\alpha . \end{array}$$

**Proof.** First, we obtain

(A60) $$\begin{array}{cc} P(\mathrm{card}U\left(X_{1}^{n}\right)\leq m_{n}) & \leq \sum_{k=1}^{m_{n}}P\left(g\left(k;X_{1}^{n}\right)\neq Z_{k}\right)=\sum_{k=1}^{m_{n}}{\left[1-P(K_{i}=k)\right]}^{n}\\ & \leq m_{n}{\left[1-\frac{m_{n}^{-\alpha }}{\zeta (\alpha )}\right]}^{n}\leq m_{n}\exp\left(-nm_{n}^{-\alpha }/\zeta \left(\alpha \right)\right), \end{array}$$

where $\zeta \left(\alpha \right):=\sum_{k=1}^{\infty }k^{-\alpha }$ is the zeta function. Put now $m_{n}=n^{1/\alpha -\epsilon }$ for an ϵ>0. It is easy to observe that $\sum_{n=1}^{\infty }P\left(\mathrm{card}U\left(X_{1}^{n}\right)\leq m_{n}\right)<\infty$. Hence, by the Borel–Cantelli lemma, we have inequality $\mathrm{card}U\left(X_{1}^{n}\right)>m_{n}$ for all but finitely many *n* almost surely. Second, we obtain

(A61) $$\begin{array}{cc} P\left(\mathrm{card}U\left(X_{1}^{n}\right)\geq M_{n}\right) & \leq \frac{n!}{(n-M_{n})!}\prod_{k=1}^{M_{n}}P\left(K_{i}=k\right)\\ & =\frac{n!}{\left(n-M_{n}\right)!{\left(M_{n}!\right)}^{\alpha }{\left[\zeta \left(\alpha \right)\right]}^{M_{n}}}. \end{array}$$

Recalling from Appendix B that $\log n!=n\left(\log n-1\right)+\log^{*}n$, where $\log^{*}n\leq \log\left(n+2\right)$ is subadditive, we obtain

(A62) $$\begin{array}{cc} \log P(\mathrm{card}U\left(X_{1}^{n}\right)\geq M_{n}) & \leq n\left(\log n-1\right)-\left(n-M_{n}\right)\left[\log(n-M_{n})-1\right]\\ & \;-\alpha M_{n}\left(\log M_{n}-1\right)+\log^{*}M_{n}-M_{n}\log\zeta \left(\alpha \right)\\ & \leq M_{n}\left[\log n-\alpha \left(\log M_{n}-1\right)-\log\zeta \left(\alpha \right)\right]+\log^{*}M_{n} \end{array}$$

by $\log n\leq \log\left(n-M_{n}\right)+\frac{M_{n}}{n}$. Put now $M_{n}=Cn^{1/\alpha }$ for a $C>e{\left[\zeta \left(\alpha \right)\right]}^{-1/\alpha }$. We obtain

(A63) $$\begin{array}{c} P\left(\mathrm{card}U\left(X_{1}^{n}\right)\geq M_{n}\right)\leq \left(Cn^{1/\alpha }+2\right)\exp\left(-\delta n^{1/\alpha }\right), \end{array}$$

where δ>0 so $\sum_{n=1}^{\infty }P\left(\mathrm{card}U\left(X_{1}^{n}\right)\geq M_{n}\right)<\infty$. Hence, by the Borel–Cantelli lemma, we have inequality $\mathrm{card}U\left(X_{1}^{n}\right)<M_{n}$ for all but finitely many *n* almost surely. Combining this result with the previous result yields equality (A58). To obtain equality (A59), we invoke Theorem A9 for the lower bound, whereas, for the upper bound, we observe that

(A64) $$\begin{array}{c} E\;\mathrm{card}U\left(X_{1}^{n}\right)\leq M_{n}+nP\left(\mathrm{card}U\left(X_{1}^{n}\right)\geq M_{n}\right), \end{array}$$

where the last term decays according to the stretched exponential bound (A63) for $M_{n}=Cn^{1/\alpha }$. ☐
